# Effectiveness of YOLO variants for small object detection in SAR images using a new dataset

**DOI:** 10.1038/s41598-025-28755-3

**Published:** 2025-11-26

**Authors:** Kinga Karwowska, Jakub Slesinski, Damian Wierzbicki

**Affiliations:** Department of Imagery Intelligence, Faculty of Civil Engineering and Geodesy, Military University of Technology, Warsaw, Poland

**Keywords:** Remote sensing, Synthetic aperture radar (SAR), Object detection, Despeckling, YOLO evolution, Civil engineering, Scientific data

## Abstract

Small object detection in SAR imagery remains challenging due to limited availability of specialized datasets. The article presents a new SAR dataset designed for small-object detection. Due to the absence of publicly available datasets dedicated to vehicle detection on satellite radar imagery, a custom dataset containing 23,644 manually labelled vehicles was created using Capella and ICEYE imagery Also the results of an extensive comparative analysis of three YOLO architectures (versions 7, 8, and 12) in the task of detecting small vehicles in radar imagery were presented. The study also considers the influence of image filtering on detection effectiveness. Experimental results provided new insights into fine-tuning YOLO architectures specifically for detecting small objects in synthetic aperture radar (SAR) images. In addition, the SIVED (SAR Image dataset for VEhicle Detection) dataset (high-resolution airborne imagery) was used in the study. Model performance was tested under various configurations and with Lee, Frost, and GammaMAP filters. Furthermore, a detailed analysis of model stability was performed. The experimental results revealed notable differences in performance among the tested models. The YOLOv8 model achieved the highest detection performance on the SIVED dataset, with an F1-score of 0.958 and mAP@[0.5:0.95] of 0.838 in the unfiltered scenario, along with high stability with respect to changes in threshold parameters. The YOLOv12 model demonstrated its best performance after Lee filtering (F1 score = 0.951, mAP@[0.5:0.95] = 0.774), indicating a greater sensitivity to the quality of the input data. On the contrary, the YOLOv7 model exhibited high sensitivity to changes in confidence thresholds, necessitating precise parameter tuning. The conducted research has shown that YOLOv8 achieves superior detection performance on satellite radar imagery samples despite not incorporating advanced self-attention mechanisms. This work contributes significantly to automatic object detection in radar images, providing practical guidelines for selecting and configuring YOLO models according to the characteristics of the SAR data.

## Introduction

Technological advancements in the field of synthetic aperture radar (SAR) have led to a rapid increase in the number of high-resolution images. The manual analysis of such data is becoming increasingly inefficient and error-prone. As a result, there is a growing demand for modern, automated methods for processing and analyzing SAR data, particularly in the context of object detection and recognition.

SAR systems have become widely adopted in civilian domains, such as critical infrastructure monitoring, urban area observation, natural disaster detection (floods, earthquakes, and landslides) and maritime traffic surveillance. The quality of the data depends on technical parameters such as antenna size and synthetic aperture length, as well as on the signal processing algorithms used (Range Doppler, Chirp Scaling, Omega-K, SPECAN^1^.

In recent years, there has been rapid growth in the commercial sector – companies such as ICEYE, Capella Space, Umbra, and Synspective are steadily expanding their constellations of SAR satellites^2^. As a result, radar data is becoming available with increasing frequency, and its resolution and quality are systematically improving.

Despite advances in the automation of SAR data analysis, object detection remains a challenging task, mainly due to the significant variability of radar signatures. In SAR systems, the signal reflected from a target is recorded as a backscatter coefficient (σ⁰), the value of which depends not only on the properties of the object itself (e.g. shape, material, orientation) but also on system parameters (wavelength, incidence angle, polarisation). The result is images in which the same objects may appear differently depending on acquisition conditions. An additional challenge is speckle noise, which results from the interference of waves reflected from multiple surfaces within a single pixel. Complex interactions between the target, system, and environment – including the influence of topography and surface roughness – lead to significant ambiguity in the appearance of the same object under different imaging conditions. As a result, detection systems must cope with large visual discrepancies and limited contrast between the object and the background.

Despite these limitations, SAR imaging has clear advantages over optical sensors – particularly its ability to image in all weather conditions and at night – and its use for detecting small objects, such as cars, has gained increasing attention. These characteristics make SAR particularly useful in low-visibility situations where electro-optical sensors often fail, such as at night, during cloud cover, or in adverse conditions. When optical data are unavailable, SAR data can aid in traffic optimization and smart city development by supporting parking occupancy monitoring and vehicle counting in urban environments. On a larger scale, analysts may infer industrial activity patterns by systematically monitoring parking areas near ports or manufacturing facilities. This provides indirect indicators of vehicle production, export volume, and economic health. Furthermore, SAR is well-suited for military and security-related applications, such as vehicle movement surveillance in contested or remote areas, border monitoring, and logistics tracking. The growing commercial availability of high-resolution SAR imagery further enables continuous, cost-effective monitoring of these scenarios, encouraging the development of robust and automated vehicle detection algorithms.

In the context of radar imaging, the detection of small objects represents a significant research challenge. Small objects – typically defined as those occupying fewer than 32 × 32 pixels or less than 10% of the image dimensions – are particularly difficult to detect, as defined in standard benchmarks such as MS COCO^3^.

**Fig. 1:**
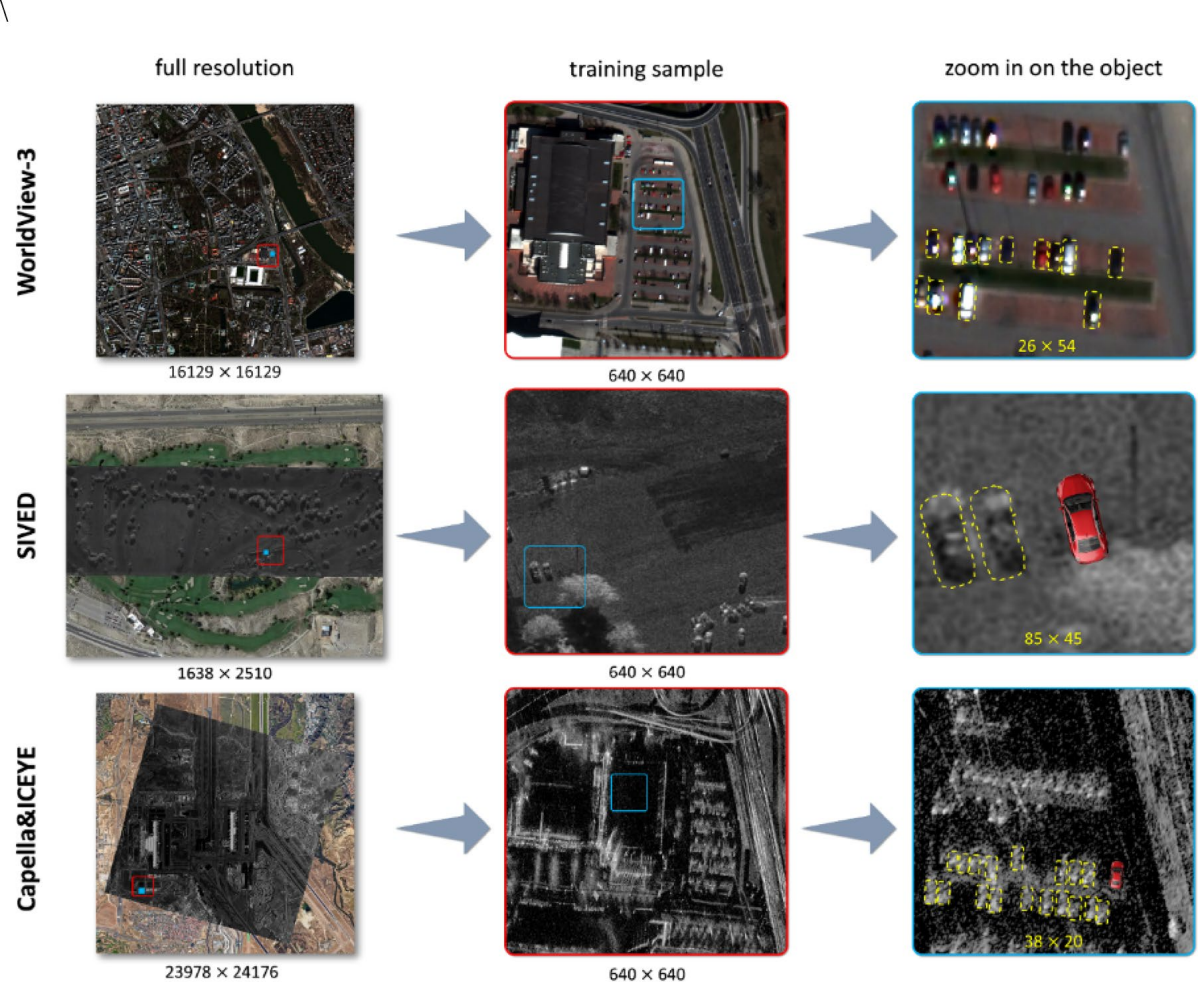
Comparison of the visibility and representation of vehicles in images: electro-optical (WorldView-3) and radar (miniSAR, ICEYE). Imagery displayed in QGIS; graphic elements prepared in Microsoft PowerPoint. WorldView-3 and ICEYE images originate from the authors’ licensed archives. The miniSAR example is from the public SIVED^30^ dataset.

Figure 1 illustrates the scale of the challenge posed by small object detection in different types of image data, especially SAR radar imagery. The first column presents the source images: a scene acquired by WorldView-3, an airborne SAR scene from the miniSAR system (as in the SIVED dataset), and a satellite SAR image acquired by an ICEYE satellite. The second column shows sample training crops of 640 × 640 pixels, typically used in detection models such as YOLO. The third column displays enlargements of individual vehicles in these data. The differences in object representation are evident: in optical images, a passenger car occupies just 26 × 54 pixels, which, despite the limited size, allows recognition of the shape and context of the object. Meanwhile, in radar data, the object’s signature contains much less information – a vehicle in the SIVED dataset occupies 85 × 45 pixels, and in high-resolution satellite SAR data only 38 × 20 pixels, which significantly limits the possibility of accurate interpretation and differentiation of objects.

It is worth noting that EO optical data (e.g., from DOTA) is much easier to interpret compared to radar images, especially those originating from satellite SAR systems.

### Related works

Object detection in Synthetic Aperture Radar (SAR) imagery has been extensively studied over the past decades. Despite technological advances, this process continues to face significant challenges – chief among them, the presence of noise characteristic of coherent radar systems.

### The impact of speckle noise on object detection

As previously mentioned, speckle noise is one of the primary obstacles in SAR image analysis. It arises from the coherent nature of imaging, where waves reflected from numerous randomly distributed scatterers within a single resolution cell interfere with each other. This interference produces the characteristic grainy appearance of SAR images. Unlike additive noise, speckle is multiplicative and nonlinear, making it difficult to filter out without sacrificing important geometric and structural features.

Many classical methods for speckle noise reduction are based on adaptive statistical filters, such as the Lee, Frost, or Gamma-MAP filters, which utilize local intensity statistics^4^. These filters improve the signal-to-noise ratio, but in many cases, they excessively smooth the image and blur critical edges, thereby reducing the precision of small object detection. In contrast, Hasnaouy and Kasapoglu^5^ introduced an averaging filter prior to feature extraction and investigated its impact on classification accuracy in SAR Automatic Target Recognition (ATR). After filtering, the accuracy of the RBF-SVM classifier increased from 97.69% to 98.43%. Researchers noted that reducing speckle noise has a significant effect on object recognition accuracy. Huang et al.^6^ proposed an innovative algorithm called Coherence Reduction Speckle Noise (CRSN), which leverages the coherence properties of SAR imaging. Wang et al.^7^ employed a convolutional neural network (CNN) with five convolutional layers and ReLU activation functions, eliminating pooling layers to preserve the original size of feature maps. Another CNN-based approach was presented by Kwak et al.^8^, who designed a model incorporating a regularization term. This component balances features extracted from both raw and filtered images, enabling the retention of essential object information even in highly noisy data. A completely different approach was proposed by Huang et al.^9^, who developed the Joint Low Rank and Sparse Multiview Denoising (JLSMD) method, combining low-rank and sparsity modeling with multiview analysis. JLSMD effectively reduces speckle noise while preserving crucial object features. The authors demonstrated that combining JLSMD with a sparse representation-based classifier (SRC) yields better results than traditional filtering methods. Chen et al.^10^ proposed an approach based on low rank and space-angle continuity.

Object detection in radar imagery using convolutional neural networks (CNNs) is currently one of the central topics in ATR research. These frameworks are typically categorized into two groups: two-stage and one-stage detectors.

Two-stage architectures, such as Faster R-CNN, first generate region proposals (Region Proposal Network), followed by classification and regression of bounding boxes. These models are characterized by high precision, especially for small object detection, at the cost of greater computational demand. Their effectiveness in SAR applications has been enhanced through techniques such as anchor tuning, hard negative mining, and adaptation to the specific nature of SAR data (e.g., DAPN)^11^,^12^. During research, scientists observed that applying transfer learning – using models pre-trained on large optical datasets (such as ImageNet) – proved to be a crucial step in adapting CNNs to SAR data when labeled data is limited. Pre-estimated network weights learned from optical images provide a better starting point than random initialization, leading to improved detection accuracy and shorter training times. Another conclusion from these studies is that anchor tuning and hard negative mining positively influence the detection of small and densely clustered targets^12,13^.

The second group consists of one-stage models, which in recent years have begun to outpace two-stage approaches. The most popular representatives include YOLO, SSD, RetinaNet, and CornerNet. These models predict object classes and locations in a single step (detecting bounding boxes and performing classification simultaneously^14^, which significantly reduces prediction time. Thanks to techniques such as attention mechanisms (e.g., in YOLO), multi-scale feature fusion (e.g., FPN in SSD), and anchor-free approaches (e.g., CornerNet), these models effectively address challenges typical for SAR imagery, such as speckle noise and varying object orientation. The latest implementations achieve precision exceeding 90% while operating in real time^15^. Architectures utilizing Feature Pyramid Networks (FPN), similar to SSD, and adaptive anchors for SAR targets reach precision levels of 94.13% at 111 frames per second^16^.

### Detection of small objects

Detecting small objects in SAR imagery remains one of the greatest challenges in AI-based object detection. The small size of targets, low image resolution, and the presence of speckle noise mean that small objects are often represented by just a few pixels. As a result, convolutional neural networks (CNNs), despite their effectiveness in conventional computer vision tasks, often struggle to detect such objects reliably.

Several approaches for detecting small objects in SAR imagery have been proposed in the literature. Zhang et al.^17^ demonstrated that quality enhancement techniques, such as multilook processing and the MUSIC algorithm, significantly increase resolution and suppress background, reducing the number of false alarms by more than threefold. Xu et al.^18^, on the other hand, proposed a modified CFAR algorithm based on the Alpha-stable distribution, which handles highly non-homogeneous clutter better than classical Gaussian and K-CFAR approaches. In the field of deep learning, Chen et al.^19^ introduced a modification of Feature Pyramid Networks (FPN), applying a k-means algorithm based on shape similarity for clustering reference boxes. This achieved detection rates over 98% in port environments.

Meanwhile, Ge et al.^20^ modified YOLOv7 – introducing Coordinate Attention (CA) into the backbone and replacing PANet with a BiFPN structure for multi-scale feature extraction – to increase the utilization of information from shallow layers and improve detection accuracy. These modifications resulted in a 3.82% accuracy improvement compared to standard YOLOv7. Sun et al.^21^ proposed a lightweight FFCLC network, integrating attention for feature fusion, depthwise separable convolutions (DWConv), and multi-scale detection. This model was tested on the SSDD dataset and achieved a mean Average Precision (mAP) of over 97%.

**Table 1:** Related works summary.

Authors	Model	Model Features	Database	Sensor		Assessment metrics				Rotatable bounding box
				Airborne	Satellite	Prec	Rec	F1-score	AP	
Wang et al.,^22^ (2025)	SVDDD	Fine-tuning of the Stable Diffusion model on SAR imagery, integrated with ControlNet for controlling vehicle position and orientation	FARAD/ MiniSAR (512 × 512)	✓	-	-	✓	-	✓	✓
Chen et al.,^23^ (2024)	GCN-YOLO	Combines a Graph Convolutional Network (GCN) with YOLO	miniSAR	✓	-	✓	✓	✓	✓	–
Han et al.,^24^ (2024)	FRA-Net	Utilization of a Spatial-Channel Reconstruction Module (SCRM) with spatial attention mechanisms	Mix MSTAR (512 × 512)	✓	-	✓	✓	✓	✓	✓
Song et al.^25^ (2023)		YOLOv5 with CAM Attention, CAM-FPN, and a Decoupled Head	WSVD (512 × 512)	–	✓	–	–	–	✓	–
Li et al.^26^ (2022)	RIRConv	Modification of convolutional layers, modulation mechanism, and integration with SSD	miniSAR	✓	–	✓	✓	✓	–	–

Table 1 summarizes and compares the proposed solutions. Analysis of the studies indicates that most research relies on airborne data (such as miniSAR, MSTAR). Only one solution^25^ utilizes satellite-acquired imagery; unfortunately, the dataset used in that study is not publicly available. Evaluation strategies vary widely – while Average Precision (AP) remains the most commonly used metric, only some authors include additional measures such as Precision (Prec), Recall (Rec), and F1-score, which enable a more comprehensive assessment of detection performance. Furthermore, our analysis shows a growing trend toward the use of rotatable bounding boxes^22,24^ in recent research.

### Datasets for small vehicle detection

With the dynamic development of Earth observation systems and the increasing number of radar satellites, the availability of SAR data is steadily growing. Consequently, recent years have seen a notable rise in publicly available SAR datasets for tasks such as detection and classification. New datasets often feature high-resolution imagery acquired from a variety of sensors and frequency bands. Table 2 presents an overview of datasets suitable for vehicle detection and classification in radar images.

**Table 2:** Compliation of databases to detect vehicles (Pol - polarization).

Dataset	Year	Sensor/Source		# Img	Img size	Res [m]	Band	Pol	Target	Labels	Application
MSTAR^27^	1995	Aerial X-band radar		14,577	128 × 128	0.3	X-band	Single	Military vehicles	Yes	Classification
SARSim^28^	2016	simulated data		21,168	139 × 139	0.1 to 0.3	X-band	Single	Military vehicles	Yes	Classification
SAMPLE^29^	2019	MSTAR + simulated data		2,732	128 × 128	0.3	X-band	Single	Military vehicles	Yes	Classification
SAR_Vehicle_Detection	2019	Airborne SAR	MiniSAR	15	various	0.1	Ku-band		Theoretically Vehicles	No	Theoretically detection
			FARAD	89		0.1	Ka-band X-band	Single			
SIVED^30^	2023	Airborne SAR synthetic slice	FARAD	1,044	512 × 512	0.1	X-band Ka-band	Single	Vehicles	Yes (OBB)	Detection
			MiniSAR			0.1	Ku-band				
			MSTAR			0.3	X-band	Single			
SARDet-100 K^31^	2024	Gaofen-3, Sentinel-1, TanDEM-X, RADARSAT-2, Alos-PALSAR, Capella, ICEYE, Kompsat-5, RISAT-1		116,598	512 × 512	0.5 to 3	L-band C-band X-band	Single, Dual	Ships, vehicles, others	Yes (AABB or OBB)	Multiclass Detection
ATRNet-STAR^32^	2025	simulated data, Airborne		194,324	128 × 128	0,12 − 0,15	X-band Ku-band	Single, Dual	Vehicles	Yes	Classification

In the field of vehicle classification, the thirty-year-old MSTAR dataset^27^, containing over 14,000 X-band radar images, continues to serve as a key benchmark. However, its limited diversity – in terms of both class variety and imaging scenarios – has led to the development of newer alternatives such as SARSim^28^ and SAMPLE^29^, which utilize both synthetic and hybrid data. Addressing the need for greater scale and realism, the ATRNet-SAR dataset was released in early 2025, comprising more than 190,000 radar images depicting realistic scenes with varied terrain, observation angles, and acquisition modes. This dataset stands out not only for its size but also for its much broader class coverage, encompassing 40 vehicle types, from small passenger cars (Mini Car) to engineering vehicles (Shovel Loader). In comparison, MSTAR includes only 10 classes. With its rich geometric and semantic representation, ATRNet-SAR has the potential to become a new research standard for vehicle classification in SAR imagery, supporting the development of models robust to changing scene and imaging conditions.

Developing datasets for vehicle detection in SAR images is significantly more time-consuming and demanding than for classification, primarily due to the requirement for precise spatial annotations. A major step forward came in 2023 with the release of the SIVED dataset – the first public SAR dataset featuring oriented bounding box (OBB) annotations. This dataset combines imagery from three sources: MiniSAR, FARAD, and MSTAR. Among more recent efforts, SARDet-100 K also stands out – a large-scale collection containing over 100,000 images from radar satellites such as Gaofen-3, Capella, ICEYE, and Sentinel-1. The diversity of sensors, spatial resolutions (0.5 to 3 m), and polarization modes (Single and Dual) enables the training of detection models resilient to varying imaging conditions.

However, it is important to note that SARDet-100 K includes a wide range of object categories – not only vehicles and ships, but also infrastructure such as bridges, airports, and ports. Another, slightly older, dataset is SAR_Vehicle_Detection, which provides data from MiniSAR and FARAD systems (Ku, Ka, and X bands), but lacks localization annotations, limiting its practical applicability.

This overview shows that most of the datasets originate from airborne platforms or synthetic sources. While some initiatives include satellite data, such as SARDet-100 K, these tend to be general-purpose collections that include multiple object categories. For small-object detection tasks, such as identifying passenger cars, SARDet-100 K may serve as a supplementary resource to more specialized datasets; however, its standalone application may be limited due to scale variability among objects.

### Motivation

As our literature review has shown, detecting small objects – such as vehicles – in SAR imagery remains a significant challenge. While numerous approaches have been proposed, including attention mechanisms, multi-scale feature fusion, and anchor optimization, a systematic evaluation of the performance of modern YOLO-family models in the context of SAR data is still lacking.

The YOLO (You Only Look Once) family of models is widely used due to its speed, extensive documentation, numerous implementations, and ongoing development. Studies in the literature have also shown that YOLO outperforms commonly used two-stage solutions, such as Faster R-CNN and Detectron 2, both in terms of detection accuracy and computational efficiency^33–35^. In our study, we focused on three versions:

- YOLOv7 – anchor-based object detection,
- YOLOv8 – anchor-free with a decoupled head,
- YOLOv12 – the latest iteration, incorporating R-ELAN and FlashAttention mechanisms.

Although researchers in the literature most often focus on evaluating successive versions of YOLO models and propose modifications aimed at improving their performance^36,37^, in our experiments we concentrated on a systematic analysis of different hyperparameter configurations. The purpose of the introduced modifications was to fully exploit the potential of each model and to identify the most effective configurations in the context of vehicle detection in SAR imagery. Our research aimed to answer the following key questions:

1. Can YOLO models be effectively applied to detect small objects in SAR data?
2. How does detection performance differ between airborne and satellite SAR imagery?
3. Does speckle noise filtering enhance detection accuracy?
4. Does FlashAttention (as in YOLOv12) improve small object detection?
5. What metrics and evaluation strategies are most appropriate for SAR-based detection tasks?

## Materials

### Capella & ICEYE vehicle dataset

Due to the lack of publicly available datasets dedicated to vehicle detection in satellite SAR imagery, we developed our own dataset. It contains 23,644 annotated vehicles in images acquired from the Capella and ICEYE satellite constellations. These data are characterized by very high spatial resolution, ranging from 0.4 m to 1 m. The dataset was divided into three subsets: training (15,755 vehicles across 913 images), validation (3,779 vehicles across 195 images), and test (4,110 vehicles across 197 images).

**Fig. 2:**
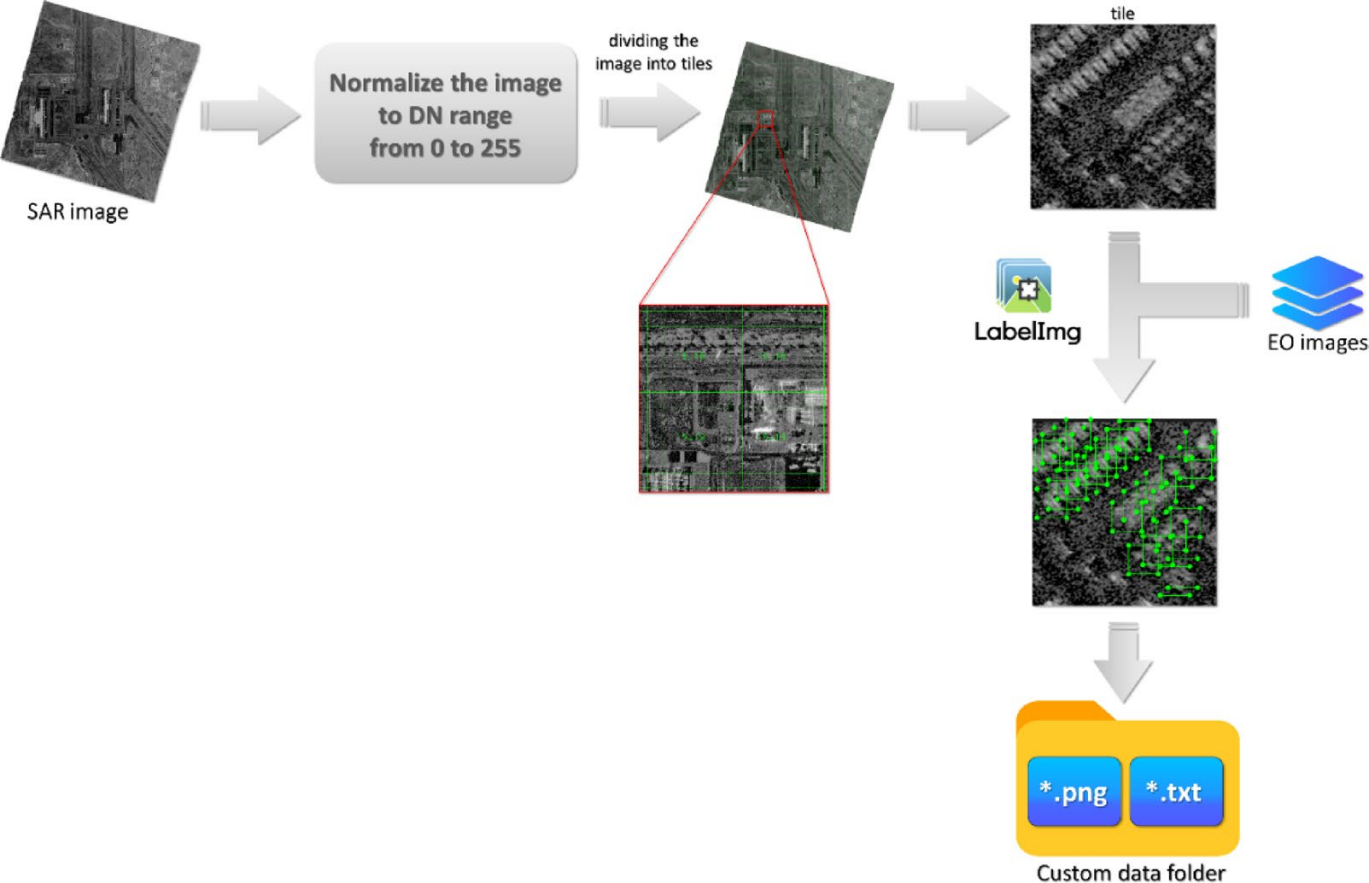
Procedure of SAR dataset preparation.

In the process of dataset preparation, the SAR images were normalized to the digital number (DN) range of 0-255, which ensured consistent intensity scaling across different scenes and enabled their further processing in the LabelImg^38^ tool. Considering that YOLO models operate on images of size 640 × 640 pixels, the SAR images were divided into smaller tiles of these dimensions. During the tiling process, an overlap of 100 pixels was applied to ensure that vehicles located at the edge of one tile were also fully visible in an adjacent tile, thereby minimizing the risk of losing objects during annotation. The generated tiles were saved in *.png format to facilitate subsequent data processing.

The next stage was the annotation process, carried out using the LabelImg tool. To reduce the number of labeling errors, additional optical Earth Observation (EO) images were used as auxiliary reference material. Finally, the annotations were saved in a format compatible with object detection models (YOLO, *.txt files). The entire dataset preparation procedure is illustrated in the Fig. 2.

### SIVED

To compare our results with those reported in the literature, we used the SIVED^30^ (SAR Image dataset for VEhicle Detection) dataset, which is one of the closest publicly available references in terms of data characteristics. It consists of 1044 image patches sourced from three different platforms: FARAD (Ka/X band), MiniSAR (Ku band), and MSTAR (X band). These images also feature very high spatial resolution, ranging from 0.1 m (FARAD, MiniSAR) to 0.3 m (MSTAR).

SIVED includes 12013 annotated vehicles, labelled using oriented bounding boxes (OBB), allowing for precise representation of object orientation and geometry, unlike traditional axis-aligned bounding boxes (AABB). The dataset is divided into three parts: training set (837 images, 9561 vehicles), validation set (104 images, 1222 vehicles), and test set (103 images, 1,230 vehicles). Due to the high annotation quality and the presence of realistic urban scenes, SIVED is widely used as a benchmark dataset in research involving rotation-aware object detection. In our work, we used this dataset as a reference, converting the oriented bounding boxes to standard rectangular format (AABB) to enable their use in conventional detection models such as YOLO. The detailed characteristics of the dataset, including year, sensor/source, number of images, image size, spatial resolution, spectral bands, polarization, target type and annotation format are summarized in Table 2.

## Methods

### Description of the applied methods

In our experiments, we utilized three versions of the YOLO model – v7, v8, and v12. These models feature diverse architectural designs, enabling an in-depth analysis of how specific components influence small object detection performance in the challenging context of SAR imagery. A detailed comparison of their characteristics is provided in Table 3.

**Table 3:** Comparison of the YOLO model variants studied.

Year	YOLOv7^39^	YOLOv8^40^	YOLOv12^41^
	2022	2023	2024
Model variant	Standard	Medium	Medium
Detection Head		Anchor-free decoupled head	Refined prediction pathways for accurate multi-scale detection; loss functions optimised for real-time performance.
Backbone	NAS-FPN	NAS-FPN-Cell	R-ELAN
Neck	PANet	PANet	FlashAttention for efficient focus on critical regions.
Attention mechanism	-	-	✓
Loss function	Decoupled loss = Complete IoU Loss + Binary Cross Entropy (BCE) (Objectness) + BCE (optional with Logits for classification) (Focal Loss can be used)	Decoupled loss = (Complete IoU Loss + Distribution Focal Loss) + BCE (Objectness) + BCE (classification)	Box loss + Class loss + DFL loss Optimised for real-time performance
Utilization	Detection 3D detection Segmentation	Detection Segmentation Pose/Keypoints	Detection Segmentation Pose/Keypoints OBB
Applied to small object detection	Supports implementation of Focal Loss	Theoretically superior to YOLOv7 due to head modification	Weight enhancement and Coordinate Attention (CA) in shallow layers
Performance (COCO)			
Params (M)	71.9	25.9	20.2
FLOPs (G)	189.9	78.9	67.5
Size (pixels)	640	640	640

YOLOv7 has gained substantial popularity in the research community due to its open-source architecture and strong performance, making it well-suited for adaptation and modification, particularly for small object detection tasks^21^. YOLOv8 introduces notable changes, discarding the anchor mechanism in favor of an anchor-free approach, designed to better detect irregular or non-standard object shapes. It also features a decoupled head – separating classification and regression branches – which enhances localization accuracy and reduces false positives for small targets. YOLOv12, the most recent version, integrates advanced improvements such as self-attention mechanisms (e.g., BiFormer Attention), an enhanced neck module for feature fusion, and modern loss functions including Distribution Focal Loss and SoftNMS. These enhancements facilitate the detection of closely spaced or partially occluded objects, which is particularly relevant for densely distributed vehicles in SAR images.

The inclusion of these three versions allows for a generational comparison: from classical anchor-based detection (YOLOv7), through anchor-free modeling (YOLOv8), to attention-augmented, optimally tuned models (YOLOv12). This comprehensive evaluation enables a systematic assessment of how architectural modifications impact small object detection in SAR data.

To further enhance vehicle detection performance in SAR imagery, we optimized selected hyperparameters for YOLOv7, YOLOv8, and YOLOv12. Default configurations are not specifically tailored to the challenges of small object detection, especially in data with high geometric complexity or low contrast, which may hinder model effectiveness.

We defined four hyperparameter configuration variants: the default setting (YOLOv7, version 0) and three modified versions (1–3), each designed to better adapt to small object detection scenarios. Table 4 provides a summary of the tested configurations, their specific parameters, and their impact on detection accuracy. Among the most impactful modifications were:

- Increased objectness weight (obj) – improved sensitivity to small objects,
- Lowered matching thresholds (iou_t, anchor_t) – enhanced anchor assignment for small targets,
- Use of Focal Loss (fl_gamma) – focused learning on difficult or ambiguous examples,
- Reduced data augmentation (mosaic, mixup, paste_in) – mitigated unrealistic distortions and occlusions.

**Table 4:** Summary of hyperparameters (where *only in YOLOv12, **only in YOLOv7).

Parameter	Hyperparameters version				Comment
	0	1	2	3	
Model training					
Box	0.05	0.01	0.1	0.1	Lower tolerance for bounding box prediction errors. Drawback: May excessively penalize the model for minor localization inaccuracies.
obj**	0.7	1	1.2	1.3	Increases the weight of detection errors. Drawback: May lead to an increased number of false positives.
iou_t**	0.2	0.1	0.05	0.05	Lower IoU threshold allows more matches for small objects. Drawback: May result in incorrect anchor assignment and degraded localization performance.
Anchor_t**	4	2	1.5	1.5	Lower threshold enables the use of smaller anchors. Drawback: Increases the risk of overfitting to random structures of similar size.
fl_gamma**	0	2	2	2	Focal Loss supports hard examples e.g., small, low-contrast objects. Drawback: Makes it harder to learn from easy examples, increasing the risk of slower convergence.
dfl*	1.5	1.5	1.5	2	Distribution Focal Loss: higher weighting focuses the model on precise bounding box localisation.
Augmentation					
hsv_s	0.7	0.3	0.3	0.3	Random saturation changes – lower values help preserve object contrast. Drawback: Reduced data diversity.
hsv_v	0.4	0.3	0.3	0.3/0.15*	Random brightness changes. Drawback: May reduce model robustness across different SAR systems.
Translate	0.2	0.1	0.1	0.1	Slight image shifts. Drawback: Decreases data variability.
Scale	0.9	0.5	0.7	0.7	Helps the model adapt to objects with variable dimensions. Drawback: Lower image quality at large scales.
Flipud	0	0	0	0.1	Vertical flipping augmentation. Drawback: May introduce unnatural or unrealistic examples.
Mosaic	1	0.5	0.8	0.8	Less distortion of small objects. Drawback: Reduces data variety.
Mixup	0.15	0	0.05	0.05	Reduces object blurring Drawback: Fewer training examples, increasing the risk of overfitting.
Paste_in**	0.15	0	0	0	Paste-in technique: inserts objects into new backgrounds – disabling it prevents occlusion of small objects. Drawback: Fewer realistic scenarios (e.g., partially occluded vehicles), which may reduce generalization.
Cutmix*	0	0	0	0.1	When the value > 0, augmentation injects patches from other images.
Erasing*	0.4	0.4	0.2	0.2	Simulates partial occlusion. High values may increase the risk of unintentionally removing the object.

Given the nature of SAR data – particularly the presence of speckle noise (as discussed in Related Works) – we also investigated the impact of noise reduction on detection quality. For this purpose, we applied commonly used adaptive filters, which operate via a two-stage process. First, they identify edge pixels by evaluating brightness variance in the local neighborhood. High variance typically indicates edge regions, which are critical for defining object boundaries.

In the second stage, smoothing is applied to non-edge regions using averaging filters. The success of this step depends heavily on selecting an appropriate window size. Smaller windows are applied in regions with high variability, while larger windows are used in homogeneous areas to increase processing efficiency. However, since window size is often fixed across the image, this can result in detail loss in heterogeneous regions^42^. Among the most widely used adaptive filters in SAR image processing are the Lee filter^43^, Gamma-MAP^44^, and Frost filter^45^.

### Evaluation of results

To evaluate the noise reduction methods and detection models, we used quality assessment metrics commonly applied in the fields of Computer Vision and Remote Sensing. A summary of the employed metrics is presented in Table 5.

**Table 5:** Summary of image quality assessment indicators and detection model assessment indicators.

Metric	Description	Goal
	Image Quality Assessment	
SSIM^46^	Measures structural similarity between two images, accounting for changes in luminance and contrast. Luminance variation is defined by the difference in mean brightness, while contrast change is measured by standard deviation: $\:SSIM\left(x,y\right)=\frac{\left(2{\mu\:}_{x}{\mu\:}_{y}+{C}_{1}\right)\left(2{\sigma\:}_{xy}+{C}_{2}\right)}{\left({\mu\:}_{x}^{2}+{\mu\:}_{y}^{2}+{C}_{1}\right)\left({\sigma\:}_{x}^{2}+{\sigma\:}_{y}^{2}+{C}_{2}\right)}$ where: x, y- compared images, $\:{\mu\:}_{x},{\mu\:}_{y}$ – mean intensities, $\:{\sigma\:}_{x}^{2}$, $\:{\sigma\:}_{y}^{2}$- variances, $\:{\sigma\:}_{xy}$ – convariance, $\:{C}_{1}$ i $\:{C}_{2}$- constant coefficients.	1
UQI^47^	Evaluates the consistency of two images in terms of correlation, luminance, and contrast. It is a precursor to SSIM and suitable for general distortion evaluation: $\:UQI\left(x,y\right)=\frac{4{\sigma\:}_{xy}{\mu\:}_{x}{\mu\:}_{y}}{\left({\sigma\:}_{x}^{2}+{\sigma\:}_{y}^{2}\right)\left({\mu\:}_{x}^{2}+{\mu\:}_{y}^{2}\right)}$	1
SCC^48^	Measures spatial linear dependence between pixel values in two images: $\:SCC\left(x,y\right)=\frac{\sum\:\left({x}_{i}-{\mu\:}_{x}\right)\left({y}_{i}-{\mu\:}_{y}\right)}{\sqrt{{\sum\:\left({x}_{i}-{\mu\:}_{x}\right)}^{2}\sum\:{\left({y}_{i}-{\mu\:}_{y}\right)}^{2}}}$	1
SAM^49^	Computes the angular difference between spectral vectors of corresponding pixels to assess spectral similarity independent of intensity: $\:SAM\left(x,y\right)={\mathrm{cos}}^{-1}\left(\frac{x\bullet\:y}{\lVertx\lVert\bullet\:\lVerty\lVert}\right)$	0°
VIFp^50^	Measures the amount of visual information shared between the reference and test image, based on perceptual models of the human visual system and information theory: $\:VIFp=\frac{{\sum\:}_{i}{\sum\:}_{k=1}^{{N}_{i}}{\mathrm{log}}_{2}\left(1+\frac{{{g}_{i}^{2}\lambda\:}_{k}^{i}}{{\sigma\:}_{n}^{2}}\right)}{{\sum\:}_{i}{\sum\:}_{k=1}^{{N}_{i}}{\mathrm{log}}_{2}\left(1+\frac{{\lambda\:}_{k}^{i}}{{\sigma\:}_{v}^{2}}\right)}$ where: $\:{\lambda\:}_{k}^{i}$- k-th eigenvalue of the signal covariance matrix in the i-th window, $\:{g}_{i}$- scaling factor, $\:{\sigma\:}_{n}^{2}$ – noise variance, $\:{\sigma\:}_{v}^{2}$ - noise variance in the internal perceptual model, $\:{N}_{i}$ - number of components in the i-th subregion.	1
ENL	Equivalent Number of Looks (ENL) estimates the signal-to-noise ratio (SNR) in SAR images. Higher ENL indicates better image quality. $\:ENL=\frac{{\mu\:}^{2}\:}{{\sigma\:}^{2}}$ where: $\:\mu\:$ - mean value of intensity in the analyzed area, $\:\sigma\:$ - variance of intensity in the analyzed area.	>
ENL ratio	Indicator of filtering effectiveness. A value greater than 1 indicates successful speckle noise reduction, while values below 1 suggest degradation: $\:{ENL}_{ratio}=\frac{{ENL}_{filtered}\:}{{ENL}_{original}}$ where: $\:{ENL}_{filtered}$ - ENL value for the filtered image, $\:{ENL}_{original}$ - ENL value for the original image.	> 1
	Detection Model Evaluation	
Precision^51,52^	Ratio of true positive detections (TP) to all detections, including false positives (FP): $\:Precision=\frac{TP}{TP+FP}$	1
Recall^51,52^	Ability of the model to detect all actual objects. Recall is defined as: $\:Recall=\frac{TP}{TP+FN}$ where FN is the number of missed objects.	1
F1-score ^51,52^	Harmonic mean of Precision and Recall. Useful when minimizing both false positives and false negatives is important: $\:F1-score=2\bullet\:\frac{Precision\bullet\:Recall}{Precision+Recall}=\frac{2TP}{2TP+FN+FP}$	1
Mean IoU^53^	For each detection, the Intersection over Union (IoU) is calculated. Mean IoU is the average IoU for all correct detections (usually IoU ≥ 0.5): $\:IoU=\frac{Area\left({B}_{pred}\cap\:{B}_{gt}\right)}{Area\left({B}_{pred}\cup\:{B}_{gt}\right)}$ $\:meanIoU=\frac{1}{N}\sum\:_{i=1}^{N}Io{U}_{i}$ Gdzie: $\:{\mathrm{B}}_{\mathrm{p}\mathrm{r}\mathrm{e}\mathrm{d}}$ – bounding box predicted by the model, $\:{\mathrm{B}}_{\mathrm{g}\mathrm{t}}$ – ground truth bounding box, Area(⋅) – operator calculating the area of a given region.	1
Average Precision (AP)^53^	Calculated as the area under the Precision–Recall curve (PR), summarizing detection performance across recall thresholds.	1

## Experiments

### Hardware description

The research activities will be carried out using a high-performance computing platform dedicated to deep learning tasks. The computing environment includes an Intel^®^ Xeon^®^ Gold 6348 processor (2.60 GHz) and 128 GB of RAM, enabling efficient processing of large datasets and memory-intensive operations. A key component of the infrastructure is the NVIDIA A100 GPU with 80 GB of VRAM, Using CUDA 12.4 and deep neural network library (CUDNN) 8.8.1 to use GPU to accelerate training.

### Experiment procedure

**Fig. 3:**
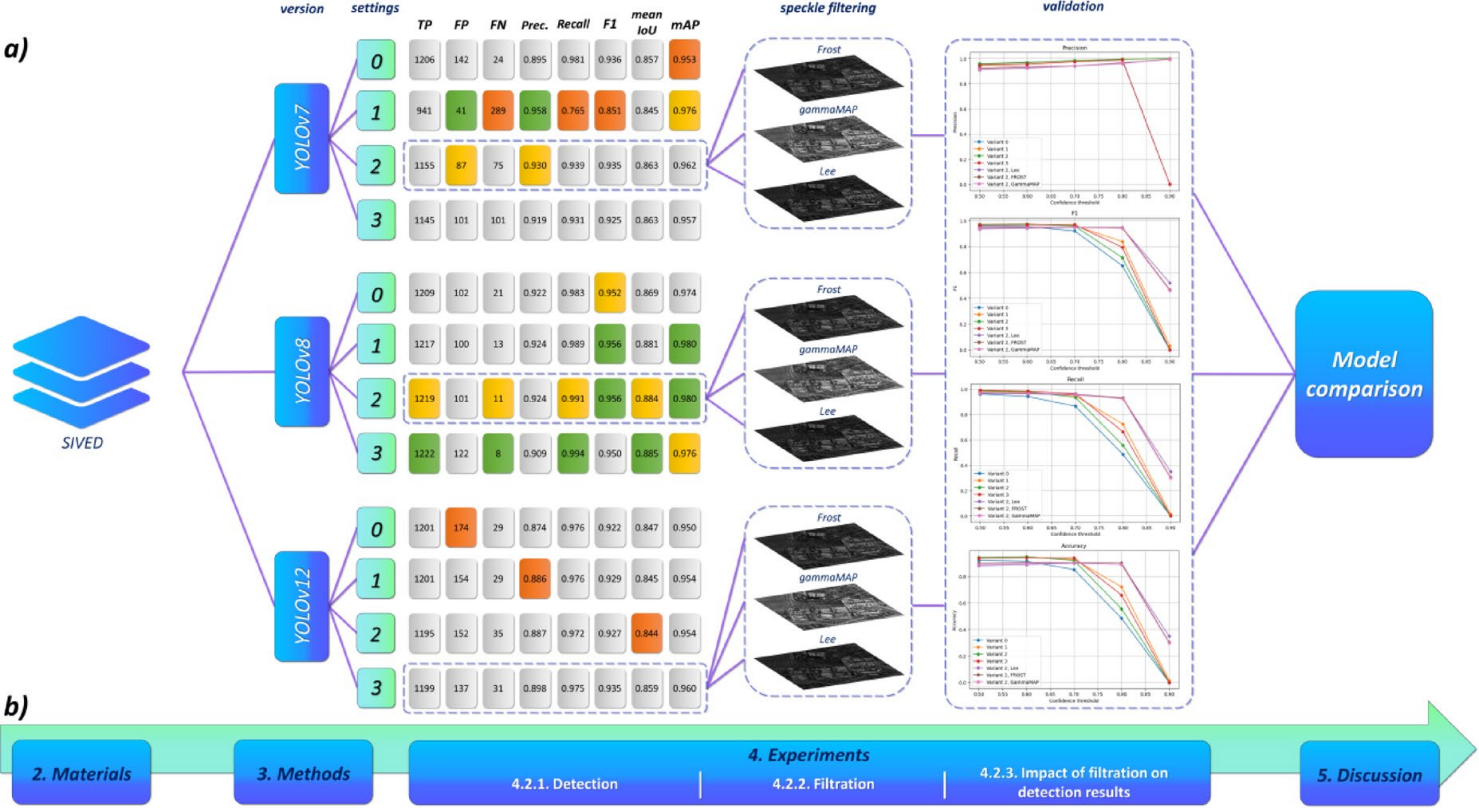
(**a**) Schematic representation of the experimental workflow using the SIVED dataset as an example, (**b**) Mapping of individual methodological steps to the respective sections of the article.

The study utilized two SAR datasets: SIVED and Capella&ICEYE, for which an analogous methodology was applied. The course of the conducted experiment (based on the SIVED dataset) is presented in Fig. 3. In the first stage of the study, various hyperparameter configurations of the YOLOv7, YOLOv8, and YOLOv12 models were tested (Methods). Based on the analysis of detection results, the best-performing configurations for each architecture were selected (Sect. 4.3.1).

In the next stage, we examined whether the application of traditional speckle noise reduction filters (Frost, GammaMAP, Lee) affects the detection quality of small objects (Sect. 4.3.2). The prepared models were then evaluated qualitatively at different confidence thresholds (Sect. 4.3.3). In Sect. 5, we conducted an extensive discussion of the obtained results.

#### Detection

To evaluate the effectiveness of vehicle detection models, we conducted a comparative study of three YOLO architectures – v7, v8, and v12 – using various training variants. The analysis included both airborne data (from the SIVED dataset) and satellite imagery, allowing for a comprehensive assessment of model performance across diverse conditions (see Tables 6 and 7).

**Table 6:**
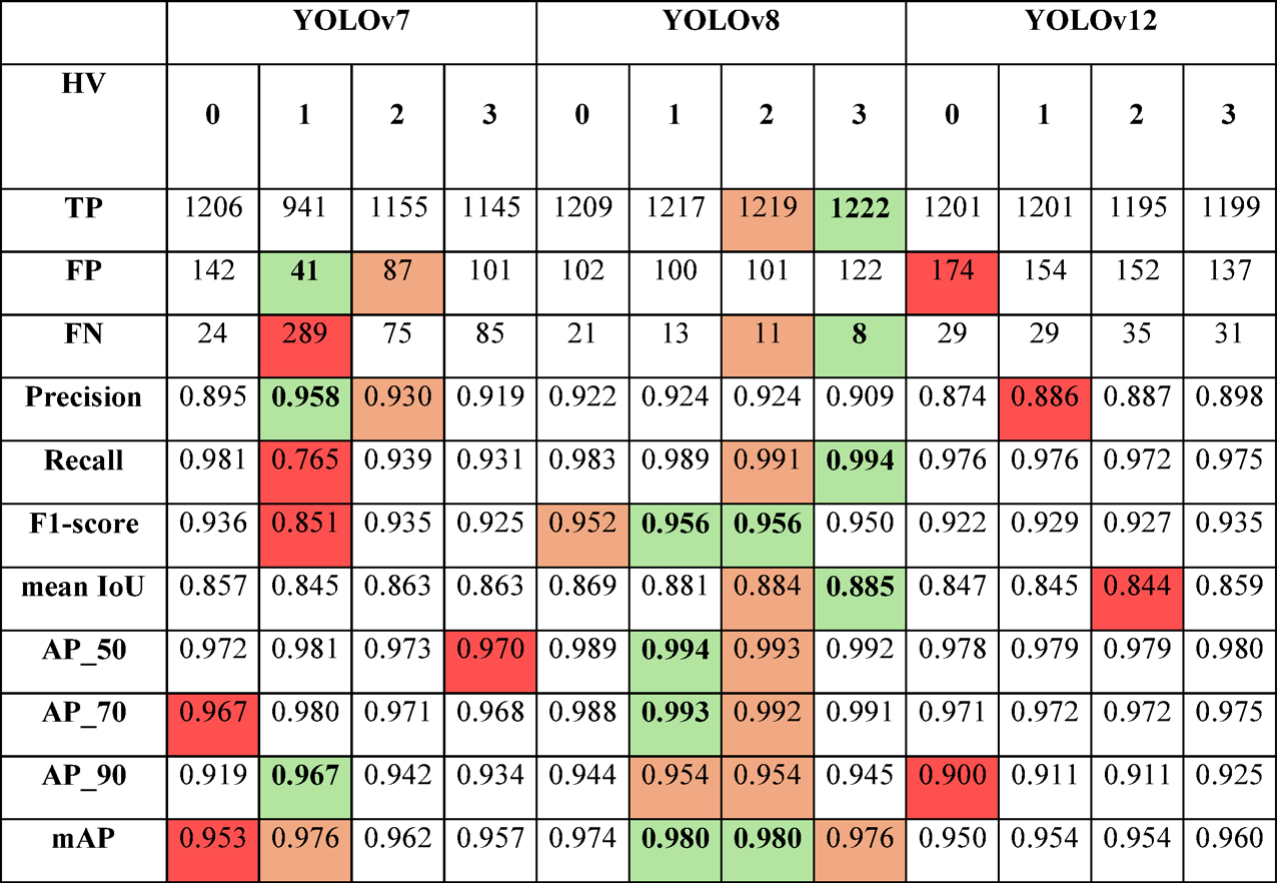
SIVED (Airborne) - qualitative assessment of detection models based on the hyperparameter variant (HV) used for training (the best results are highlighted in green, the second-best in orange, and the lowest in red).

**Table 7:**
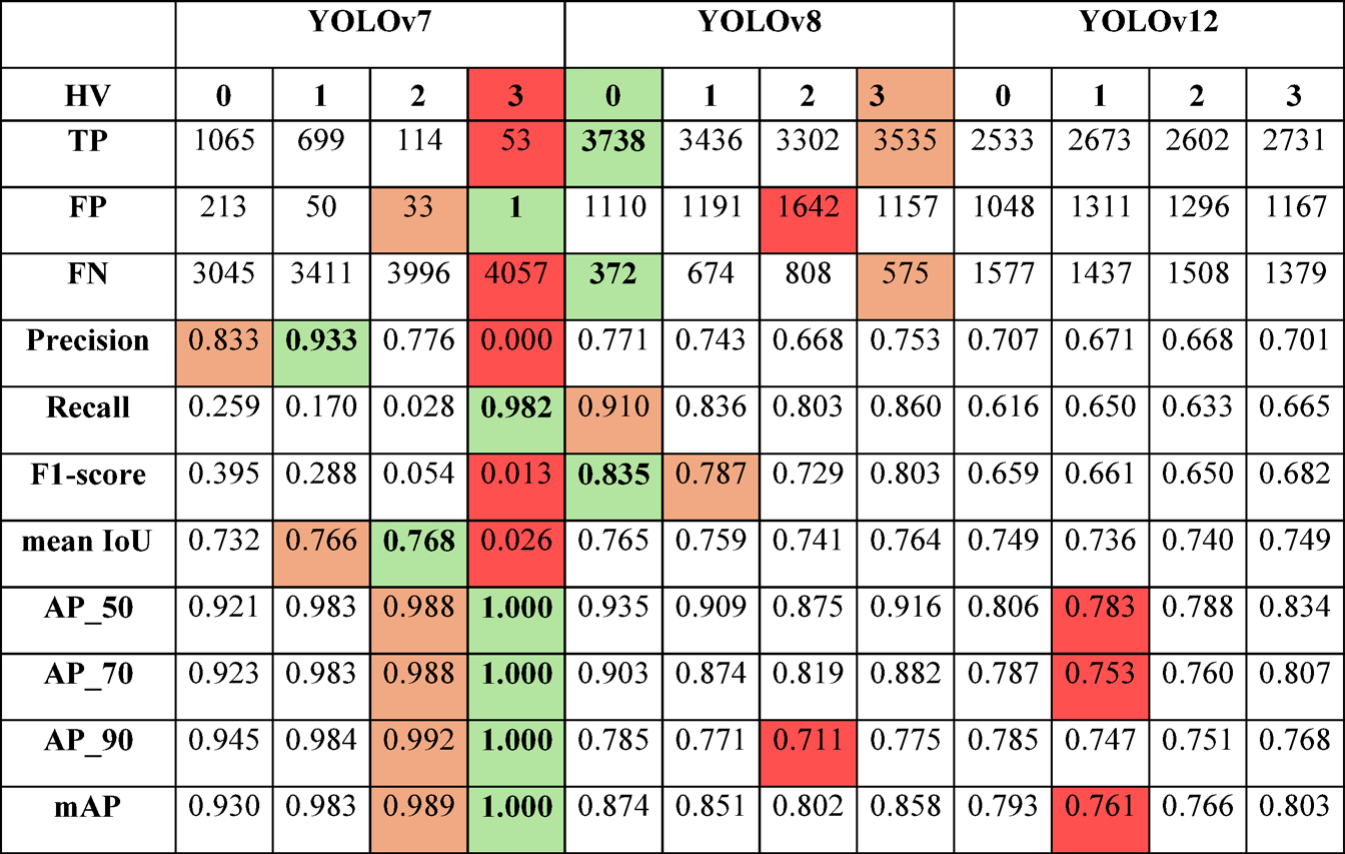
Capella&ICEYE (Satellite) - qualitative assessment of detection models based on the hyperparameter variant (HV) used for training (the best results are highlighted in green, the second-best in orange, and the lowest in red).

The YOLOv8 model achieved the highest effectiveness among all compared architectures. Regardless of the training variant, it consistently achieved high F1-scores (up to 0.956), Recall (up to 0.994), and mean IoU (up to 0.885), while maintaining low false positive and false negative rates. Particularly noteworthy are its high AP values at a strict IoU threshold (AP_90 > 0.95), indicating strong alignment between predicted bounding boxes and ground truth annotations. Variants 1 and 2 yielded only marginal improvements over the baseline (variant 0), suggesting that YOLOv8 is relatively insensitive to hyperparameter tuning and performs well even under standard configurations.

YOLOv12 also demonstrated strong performance. Its best results were achieved with variant 4 (mAP = 0.960, AP_90 = 0.925), while F1-scores across all variants ranged from 0.922 to 0.935. Although this model proved effective, it did not surpass YOLOv8. However, it showed greater robustness to hyperparameter changes compared to YOLOv7.

**Fig. 4:**
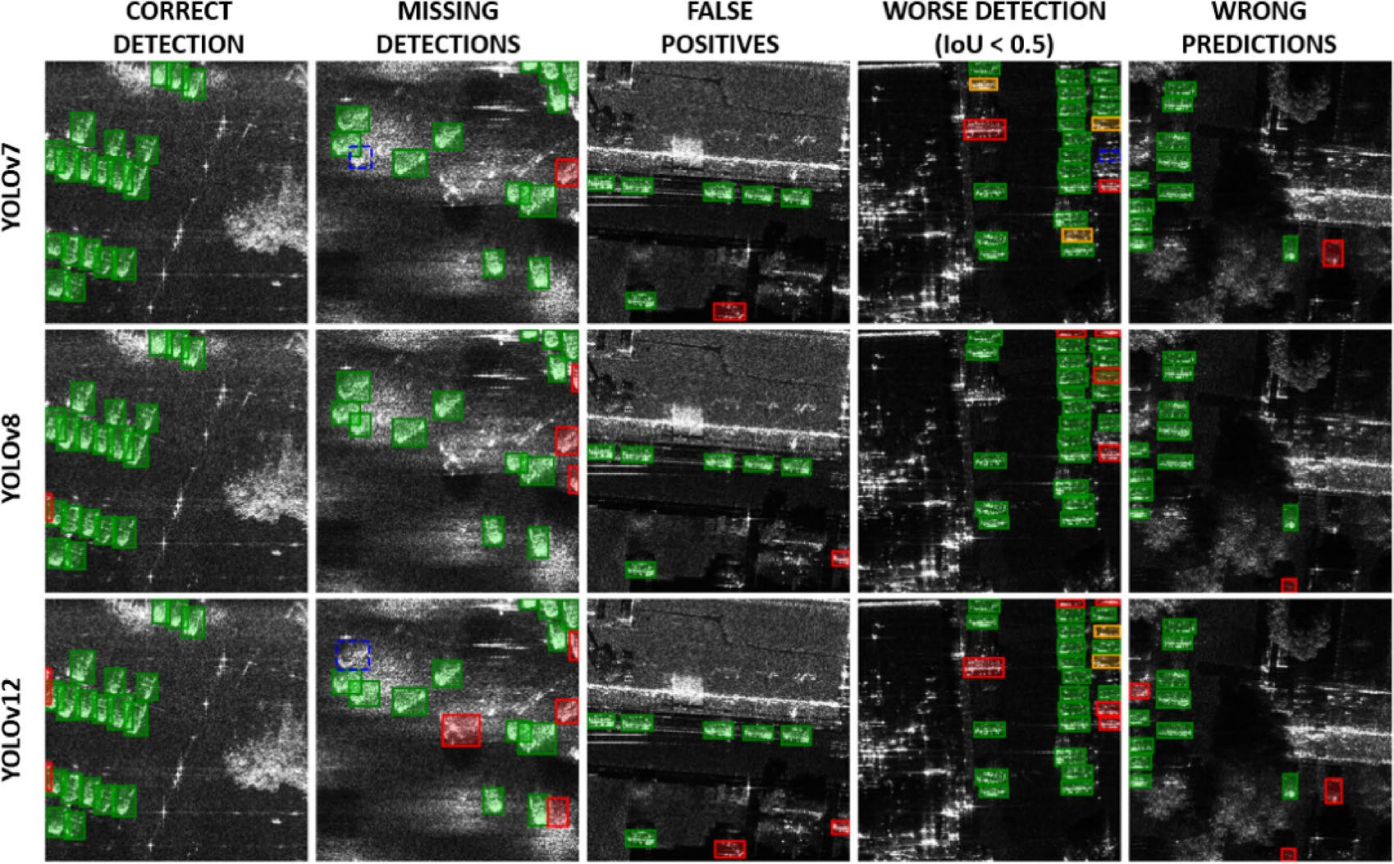
Examples of detections on the SIVED dataset. Correct detections (IoU ≥ 0.5) are shown in green, detections with IoU < 0.5 in orange, false positives in red, and missed detections (false negatives) in blue.

YOLOv7 exhibited significant sensitivity to training variants. The weakest performance was observed in variant 1 (Recall = 0.765, F1 = 0.851), while the best was in variant 2 (mAP = 0.972, F1 = 0.935). Figure 4 presents examples of vehicle detection using various YOLO models.

In the second part of the study, we assessed detection quality under more challenging conditions – specifically, satellite imagery with significantly smaller vehicle representations. Experimental results confirmed that YOLOv8 remained the most effective model for small object detection, achieving the highest scores in F1, mAP, and AP_90. Notably, its performance remained stable across all training variants, reinforcing its suitability for such tasks.

**Fig. 5:**
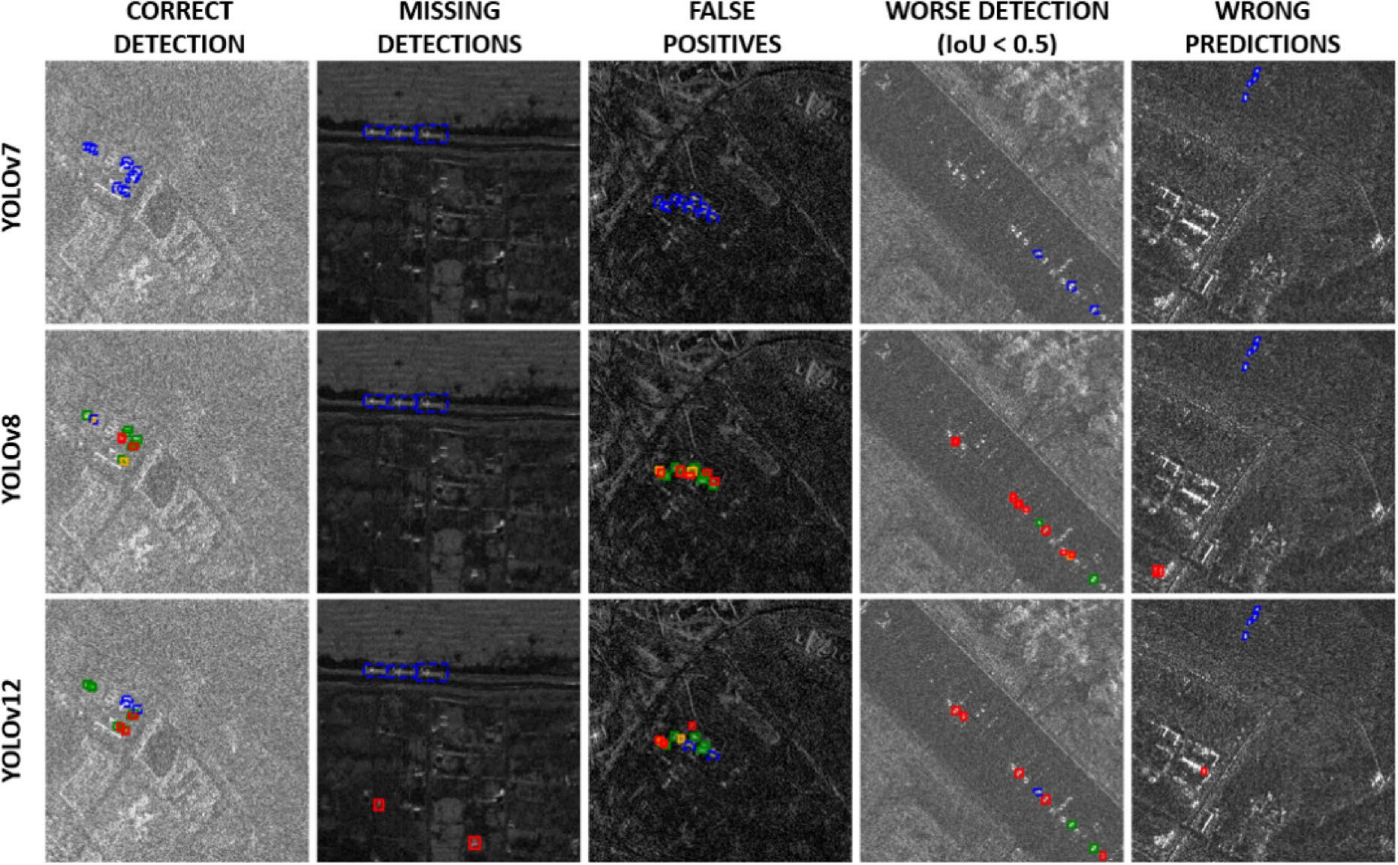
Examples of detections on the Capella&ICEYE dataset. Correct detections (IoU ≥ 0.5) are shown in green, detections with IoU < 0.5 in orange, false positives in red, and missed detections (false negatives) in blue.

YOLOv12 also showed good stability, although its lower Recall indicated a higher miss rate. YOLOv7, despite good results on airborne data, performed poorly on satellite imagery. Its limited sensitivity and strong reliance on specific hyperparameter configurations render it unsuitable for small object detection in satellite SAR imagery. Figure 5 presents examples of vehicle detection using various YOLO models.

#### Filtration

To evaluate the impact of speckle noise filtering on SAR image quality, we compared three classical filters: Frost, gamma-MAP, and Lee. Their effectiveness in preserving structural features (object detail retention) and reducing noise was assessed using five quality metrics across two radar datasets: SIVED and Capella & ICEYE.

**Table 8:** Average values of quality assessment metrics for the test sets of the SIVED and Capella&ICEYE datasets (^1^window size: 3, damping factor: 0.6, ^2^window size: 3, variance: 0.5, ^3^window size: 3, equivalent number of looks: 1, noise variance ratio: 0.25).

SIVED						
	SSIM	UQI	SCC	SAM	VIFp	ENL ratio
Frost^1^	0.766	0.962	0.793	0.224	0.382	1.361
GammaMAP^2^	0.782	0.919	0.690	0.271	0.283	0.877
Lee^3^	0.548	0.932	0.199	0.318	0.262	1.489
Capella&ICEYE						
	SSIM	UQI	SCC	SAM	VIFp	ENL ratio
Frost^1^	0.886	0.994	0.862	0.097	0.466	1.426
GammaMAP^2^	0.781	0.987	0.503	0.145	0.297	1.284
Lee^3^	0.758	0.988	0.566	0.141	0.343	1.651

**Fig. 6:**
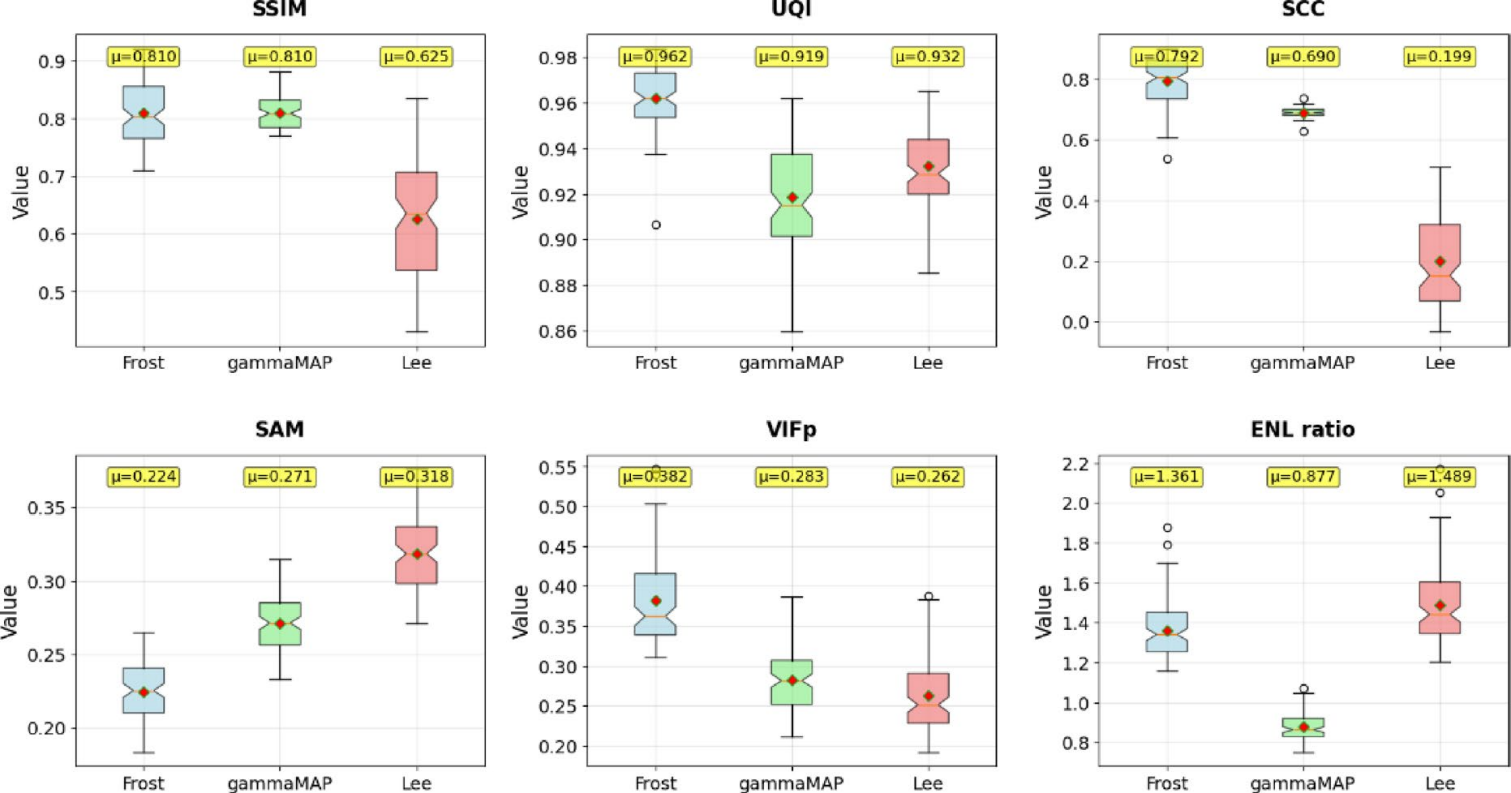
Comparison of selected quality indicators for filters: Frost, gammaMAP and Lee for the SIVED dataset.

A comparative analysis of the three filters applied to a set of 103 SAR images from the SIVED collection (Fig. 6; Table 8) revealed notable differences in performance. The Frost filter consistently delivered the best results in four out of five evaluated metrics (SSIM = 0.766, UQI = 0.962, SCC = 0.793, VIFp = 0.382), while also demonstrating the highest stability – its box plots were the most compact, indicating consistent and repeatable outcomes. Interestingly, ENL ratio analysis reveals a paradox: although the Lee filter produced the weakest scores in structural metrics, it achieved the highest ENL ratio (1.489), indicating the most effective speckle noise reduction. The Frost filter achieved moderate ENL improvement (1.361), while gamma-MAP was the only method to worsen ENL (0.877 < 1), suggesting inadequate noise suppression despite good structural preservation. The gamma-MAP filter ranked second overall, with slightly lower metric scores, but still demonstrated solid operational stability and achieved the best result in the SAM metric (0.271). In contrast, the limitations of the Lee filter were particularly apparent – it showed clearly lower median and mean values for SSIM, UQI, SCC, and VIFp, along with greater variability (wider boxes and more outliers). This was especially evident in SCC (0.199) and VIFp (0.262), where it significantly underperformed relative to the other filters. This contrast – between the Lee filter’s high ENL ratio and its low structural scores – highlights a common dilemma in SAR image processing: stronger noise reduction often compromises important structural details. Overall, the Frost filter provides the best balance between noise suppression and preservation of original image features, while the Lee filter, although effective in denoising, delivers suboptimal performance in retaining image structure.

**Fig. 7:**
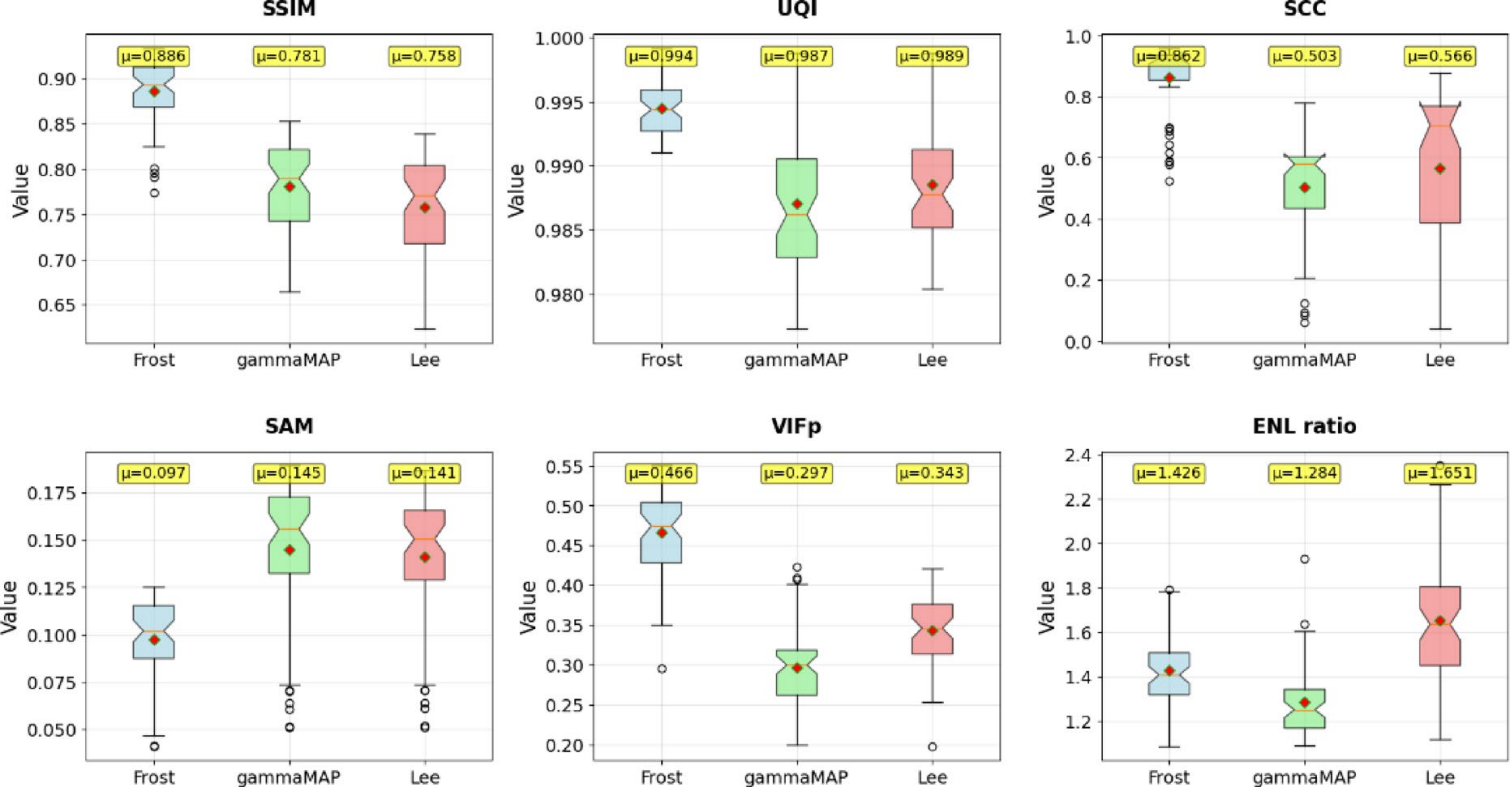
Comparison of selected quality indicators for filters: Frost, gammaMAP and Lee for the Capella&ICEYE dataset.

Applying the same filters to a dataset of 197 SAR images from Capella & ICEYE (Fig. 7; Table 7) further confirmed the superiority of the Frost filter, which outperformed across all five structural metrics (SSIM = 0.886, UQI = 0.994, SCC = 0.862, SAM = 0.097, VIFp = 0.466). In terms of ENL ratio, all three filters improved performance on this dataset (ENL > 1), with Lee again showing the highest value (1.651), followed by Frost (1.426) and gamma-MAP (1.284). A noticeable improvement was observed in gamma-MAP’s noise suppression capabilities, contrasting with its underperformance on the SIVED data. Compared to the SIVED set, the differences between the filters were even more pronounced – Frost showed significantly higher SSIM (0.120) and SCC (0.069) values. Meanwhile, gamma-MAP, which previously ranked second, showed a decline in performance, especially in SCC (0.503), though it still maintained relatively good scores in UQI (0.987) and VIFp (0.297). As with the SIVED dataset, the Lee filter remained the weakest in structural preservation, although on Capella & ICEYE data it demonstrated slightly better stability – its box plots were more compact, suggesting less erratic performance for this particular type of SAR imagery. The overall higher ENL improvements observed in the Capella&ICEYE dataset may stem from its image characteristics – higher baseline noise levels or more homogeneous regions – which make it easier for the filters to distinguish and suppress speckle noise. The increased advantage of the Frost filter in this dataset reinforces the notion that filtering strategies should be adapted to the specifics of each SAR system. ENL ratio analysis underscores the importance of balancing noise reduction with structural fidelity: the Frost filter offers the most favorable trade-off, whereas the Lee filter emphasizes denoising at the cost of spatial detail. Examples of the filtering results are presented in Table 9.

**Table 9:**
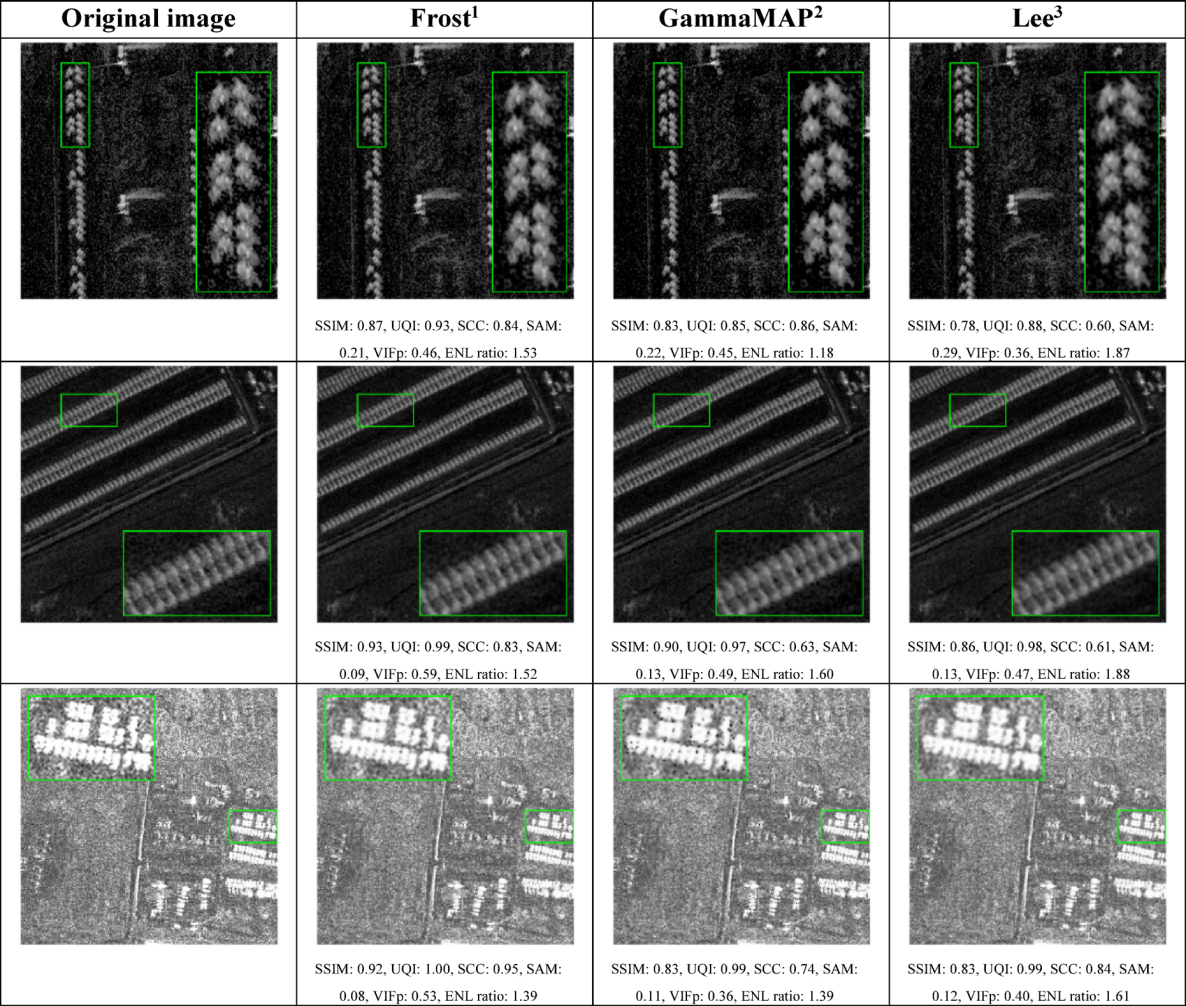
Examples of filtering results on the Capella&ICEYE dataset along with quality assessment metrics (^1^window size: 3, damping factor: 0.6, ^2^window size: 3, variance: 0.5, ^3^window size: 3, equivalent number of looks: 1, noise variance ratio: 0.25).

#### Impact of filtration on detection results

To evaluate the impact of speckle noise filtering on vehicle detection performance, we selected the best hyperparameter configurations for each of the analyzed YOLO architectures. Based on detection performance metrics (Table 10), the following configurations were used for further evaluation on the SIVED dataset:

- YOLOv7: variant 2 – characterized by a low number of false detections and high recall,
- YOLOv8: variant 2 – achieved an F1-score of 0.956, very high AP values (up to 0.994), and a mean IoU of 0.885, with one of the lowest false negative counts (FN = 11),
- YOLOv12: variant 3 – yielded the highest F1-score (0.920) and mAP (0.960) within this architecture, offering a strong balance between precision (0.898) and recall (0.975).

A similar evaluation was conducted on satellite imagery from the Capella and ICEYE constellations. Based on metric analysis (Table 8), the selected configurations were:

- YOLOv7: variant 0 – achieved the highest precision within this architecture. While recall was low due to a high number of false negatives, precision was exceptionally high, indicating cautious predictions with few false positives,
- YOLOv8: variant 2 – attained the best F1-score (0.803) for this model, with precision of 0.753, recall of 0.860, and strong mAP and mean IoU scores,
- YOLOv12: variant 3 – achieved the highest F1-score (0.682) within this version.

Table 10 Presents a comparative analysis of YOLOv7, YOLOv8, and YOLOv12, incorporating three preprocessing filters: Lee, Frost, and gammamap (GM). The evaluation covered both aerial (SIVED) and satellite (Capella&ICEYE) SAR imagery.

**Table 10:** Qualitative evaluation of detectors on sets after filtering.

	SIVED (Airborne)									Capella&ICEYE (Satellite)								
	YOLOv7			YOLOv8			YOLOv12			YOLOv7			YOLOv8			YOLOv12		
	Lee	Frost	GM	Lee	Frost	GM	Lee	Frost	GM	Lee	Frost	GM	Lee	Frost	GM	Lee	Frost	GM
TP	1155	1162	1164	1196	1197	1199	1202	1199	1189	2187	2191	1741	2783	2862	2874	2796	2829	2668
FP	109	101	98	144	124	140	156	145	148	419	429	328	1264	1117	1313	1092	1199	1046
FN	75	68	66	34	33	31	28	31	41	1923	1919	2369	1327	1248	1236	1314	1281	1442
Precision	0.914	0.920	0.922	0.893	0.906	0.895	0.885	0.892	0.889	0.839	0.836	0.842	0.688	0.719	0.686	0.719	0.702	0.718
Recall	0.939	0.945	0.946	0.972	0.973	0.975	0.977	0.975	0.967	0.532	0.533	0.424	0.677	0.696	0.699	0.680	0.688	0.649
F1	0.926	0.932	0.934	0.931	0.939	0.933	0.929	0.932	0.926	0.651	0.641	0.564	0.682	0.708	0.693	0.699	0.695	0.682
mean IoU	0.868	0.864	0.863	0.868	0.869	0.865	0.864	0.861	0.861	0.747	0.746	0.740	0.745	0.747	0.745	0.746	0.749	0.747
AP_50	0.975	0.971	0.969	0.983	0.986	0.988	0.983	0.980	0.981	0.905	0.894	0.914	0.826	0.835	0.813	0.834	0.827	0.846
AP_70	0.971	0.967	0.966	0.978	0.982	0.982	0.978	0.975	0.976	0.898	0.888	0.910	0.796	0.807	0.782	0.806	0.798	0.821
AP_90	0.939	0.936	0.937	0.936	0.944	0.937	0.923	0.925	0.924	0.894	0.893	0.918	0.755	0.774	0.747	0.779	0.762	0.788
mAP	0.962	0.958	0.957	0.965	0.971	0.969	0.961	0.960	0.960	0.899	0.892	0.914	0.792	0.805	0.781	0.806	0.796	0.818

To better assess detection performance, metrics were calculated across various confidence thresholds. This allowed for evaluation of each model’s stability and the identification of threshold ranges where precision and recall were optimally balanced.

For YOLOv7 on the SIVED dataset, variant 2 without filtering performed best, yielding an F1-score of 0.93 and mAP@[0.5:0.95] of 0.75. On satellite data, variant 1 without filtering gave the highest mAP@[0.5:0.95] (0.72), but with very low recall - indicating rare but highly accurate detections. Filtering had limited benefit, occasionally improving mAP slightly but often reducing recall and the F1-score.

**Fig. 8:**
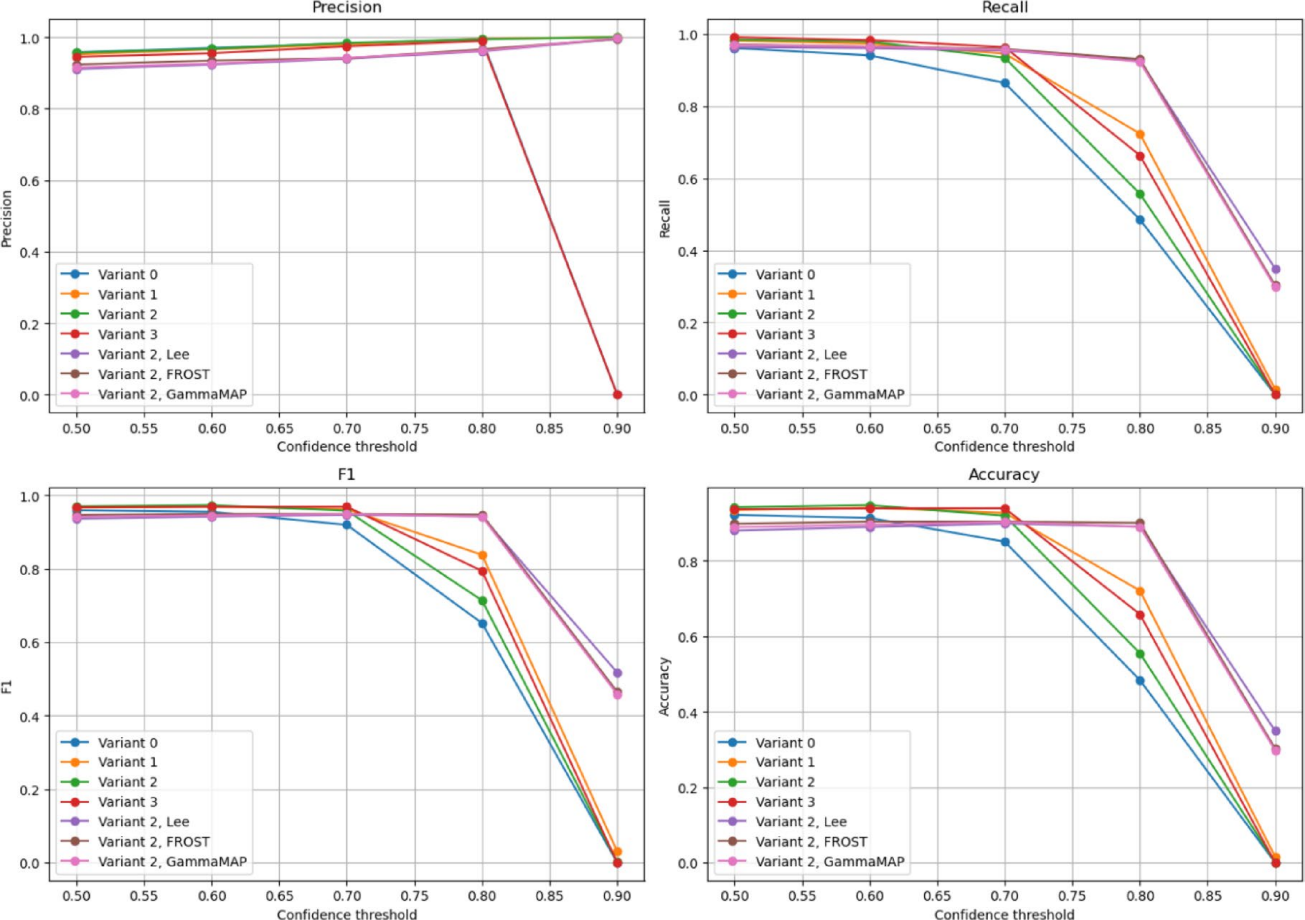
Impact of confidence threshold on vehicle detection performance for different training variants and filtering methods. Plots show Precision, Recall, F1-score, and Accuracy for YOLOv8 on the SIVED dataset across selected configurations (Variants 0–3) and filters (Lee, Frost, GammaMAP).

YOLOv8 demonstrated the highest performance on the SIVED dataset in variant 2 without filtering at a confidence of 0.7, achieving an F1-score of 0.958 and mAP@[0.5:0.95] of 0.838, indicating a solid balance between precision (0.984) and recall (0.934) (Fig. 8). Filtering did not significantly enhance detection; while some increases in mAP@0.5 were observed (e.g., 0.982 with Lee at confidence = 0.8), these were accompanied by major drops in recall and F1-score (e.g., 0.555). On satellite data, performance dropped substantially. Variant 2 (no filtering, confidence = 0.7) gave mAP@[0.5:0.95] of 0.701, but with very low recall (0.110) and F1-score (0.198). Though precision remained high (1.0), many objects were missed. Again, filtering occasionally improved specific metrics, but not overall detection effectiveness.

In contrast, YOLOv12 performed best in variant 4 with the Lee filter applied. On the SIVED dataset, this setup achieved the highest values: F1-score = 0.951, mAP@0.5 = 0.869, and mAP@[0.5:0.95] = 0.774. On satellite imagery, the same variant (with Lee, confidence = 0.7) achieved lower performance: F1-score = 0.048, mAP@0.5 = 0.639, and mAP@[0.5:0.95] = 0.560 – highlighting the persistent difficulty of detecting small objects.

**Fig. 9:**
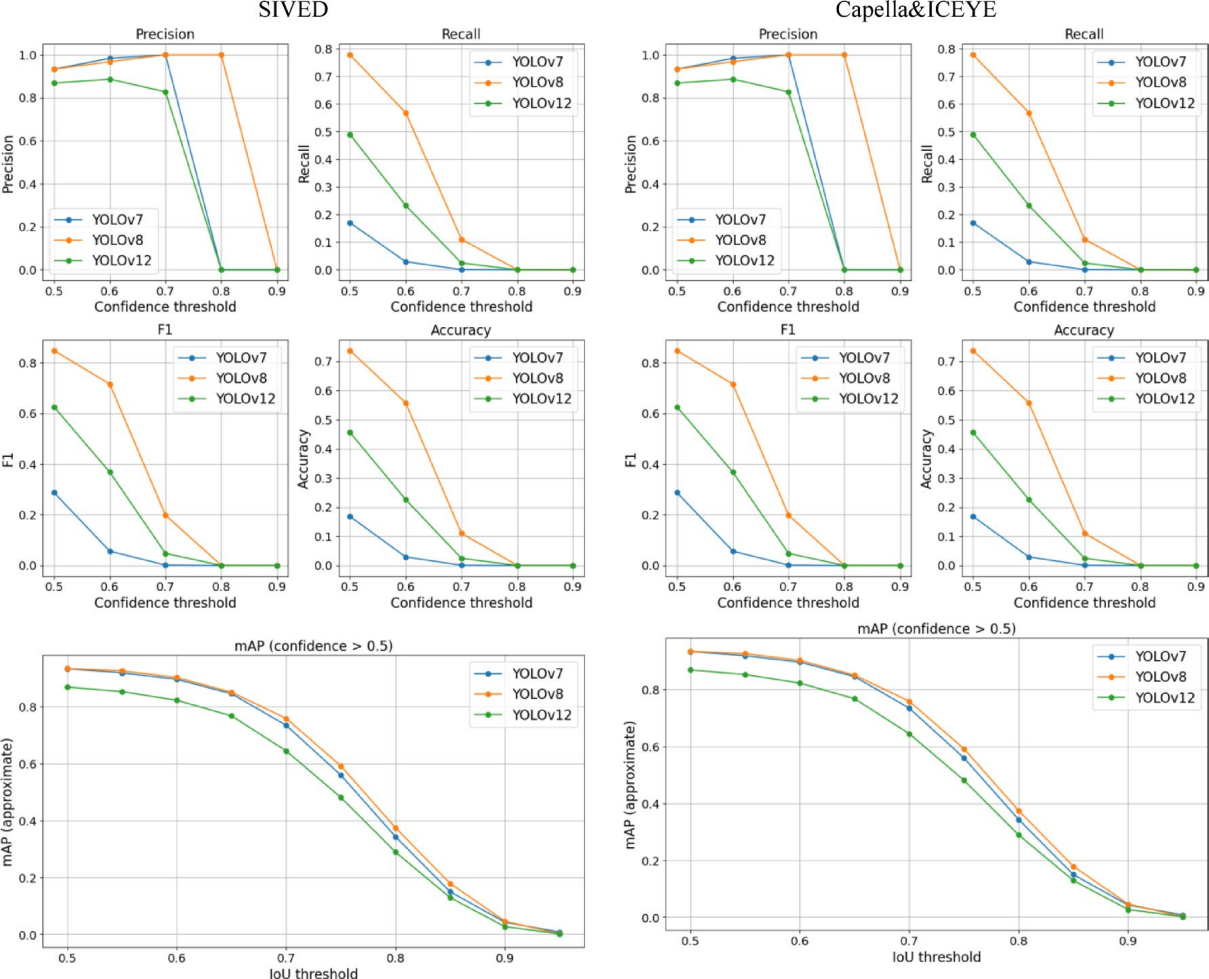
Performance comparison of YOLOv7, YOLOv8, and YOLOv12 on the SIVED (left) and Capella&ICEYE (right) datasets across different confidence and IoU thresholds.

A comparative analysis of the models revealed significant differences in robustness to threshold changes and input data quality. As shown in the plots (Fig. 9), YOLOv8 demonstrated the highest stability across varying IoU and confidence thresholds. It consistently achieved top mAP@[0.5:0.95] and F1-score values, with balanced precision and recall – indicating strong generalization and robustness to noise.

YOLOv7, however, was highly sensitive to confidence thresholds. While precision increased with higher thresholds, recall dropped rapidly, reducing the F1-score to zero at confidence = 0.8. This architecture requires careful tuning. YOLOv12 performed well in its base version, but showed significant improvement after filtering. The gain in F1-score and mAP following application of the Lee filter suggests that this model benefits from a simplified, denoised input – indicating greater dependence on signal clarity.

In summary, YOLOv8 proved to be the most effective architecture for SAR-based vehicle detection. Despite lacking advanced self-attention mechanisms, it showed resilience to speckle noise and performed well without preprocessing. In contrast, YOLOv12 reached peak performance only after filtering with the Lee filter – which, paradoxically, performed the worst in classical image quality assessments. A possible explanation is that the Lee filter simplifies the background and enhances consistency, which may improve feature extraction in deeper layers of the model. Although it degrades local structures, the uniform intensity profile may better align with YOLOv12’s internal representations, leading to improved small object detection.

To assess the effectiveness of the proposed detection models, their performance was compared with results reported in other studies. Since the SIVED dataset is relatively new, the number of existing publications using it remains limited. Additionally, most prior work fully leverages SIVED by performing object detection using oriented bounding boxes (OBB), which account for object rotation. Table 11 compares our YOLOv7, YOLOv8, and YOLOv12-based models with those reported by other research teams. It is important to note that all our models operate with classic axis-aligned bounding boxes (AABB), without incorporating rotation.

**Table 11:** Performance comparison of YOLO-based models (YOLOv7, YOLOv8, YOLOv12) and state-of-the-art rotation-aware detectors in the task of small object detection on the SIVED dataset (* without Bbox rotation).

Network	Recall	Precision	mAP	mAP_75_	mAP_50_
YOLOv7*	0.765	0.958	0.740	0.840	0.958
YOLOv8*	0.985	0.957	0.810	0.923	0.957
YOLOv12*	0.974	0.915	0.738	0.827	0.914
OrientedRepPoints^30^	0.980	0.951	0.601	0.707	0.991
GlidingVertex^30^	0.981	0.957	0.555	0.509	0.977
RotatedFasterR-CNN^30^	0.978	0.956	0.531	0.501	0.978
KLD^30^	0.980	0.933	0.575	0.645	0.979
RotatedRetinaNet^30^	0.975	0.927	0.531	0.509	0.978
S2A-Net^30^	0.975	0.909	0.555	0.573	0.977
RotatedFCOS^30^	0.889	0.965	0.504	0.481	0.956
RoITransformer^30^	0.956	0.844	0.375	0.169	0.935
Han et al.^24^	0.995	0.982	–	–	0.992
DenSe-AdViT^54^	0.978	–	0.925	–	–
ViTDet^55^	0.971	–	0.913	–	–
SwinTransformer^56^	0.976	–	0.901	–	–
Oriented-RCNN^57^	0.981	–	0.902	–	–

The best result in this comparison was achieved by the model proposed by Han et al.^24^, which reached exceptionally high Recall and Precision, as well as an AP of 0.992. Close behind was the YOLOv8 model, which – despite not incorporating rotation – achieved the second-highest mAP among all compared methods. YOLOv12 also proved highly competitive, outperforming many advanced rotated bounding box detectors.

These findings confirm that YOLO architectures are a viable alternative to more complex, rotation-aware methods, especially in real-time applications and systems with hardware constraints.

It is worth emphasizing that all presented results pertain to the SIVED dataset, based on airborne data with high spatial resolution and good radiometric quality.

## Discussion

This study evaluated three architectures from the YOLO family – v7, v8 and v12 – for the task of detecting small vehicles in SAR data acquired from both airborne and satellite sources. The data used were obtained from various sensors with differing spatial resolutions, frequency bands, and polarizations. The results indicate that both the choice of neural network architecture and careful data preparation significantly affect the quality of small object detection in radar imagery. Moreover, the effectiveness of these strategies depends on the spatial resolution of the data and the intended application of the model.

### Speckle noise and model architecture

An analysis of the impact of speckle noise and its reduction on the detection of small objects in SAR images revealed clear differences in model behavior. YOLOv7 and YOLOv8 achieved the highest detection metrics (F1-score, mAP) on unfiltered data, indicating strong tolerance to speckle noise. This is possibly due to their use of classical feature propagation mechanisms (PANet) and primarily local convolutional operations. For these models, local intensity gradients and fine texture details – features degraded by noise-reducing filters – are crucial.

In contrast, YOLOv12 delivered its best results after applying the Lee filter, despite this filter yielding the lowest scores in traditional image quality metrics (SSIM, VIFp). YOLOv12 relies on more advanced information processing mechanisms, including Residual Efficient Layer Aggregation Networks (R-ELAN) and FlashAttention, which enable global feature aggregation and capture long-range dependencies across image regions. In this case, the contrast between the object and the background is more important than local edge details. The Lee filter’s strong background smoothing benefits the attention mechanism.

### Object detection in satellite imagery

Recent years have seen a rapid increase in the availability of high-resolution SAR imagery, enabling detailed scene analysis suitable for detecting and recognizing very small objects. Despite this progress, most research on object detection in SAR images has focused on larger targets such as ships or airplanes. Widely used datasets – including OpenSARShip-2.0, SSDD, SADD, SAR-Ship-Dataset, and AIR-SARShip-1.0/2.0 – are primarily designed for ship or aircraft detection. These datasets generally offer relatively low spatial resolutions ranging from about one meter to several dozen meters. However, the growing availability of sub-meter resolution imagery is creating opportunities to develop new, more diverse datasets, which may significantly accelerate research on small object detection in SAR imagery.

### The impact of training data diversity on detection effectiveness

This study observed that training the YOLOv7 model (which performed the worst in experiments) with an expanded dataset that included not only small vehicles but also other object classes – such as airplanes and vehicle clusters where individual signatures are often indistinguishable – had a positive effect on detection performance (see Table 12).

**Table 12:** Comparison of YOLOv7 (hyperparameters version − 2) detection performance for small objects using the Capella&ICEYE dataset and an extended dataset including additional object classes.

	TP	FP	FN	Precision	Recall	F1-score	Mean IoU	mAP
Dataset								
Capella&ICEYE	114	33	3996	0.776	0.028	0.054	0.768	0.989
Capella&ICEYE (Multi-Class)	1480	84	2630	0.946	0.360	0.522	0.758	0.978

This comparison shows that the model trained with various object types – not just vehicles – achieved the highest values for key metrics, including mAP, Precision, and mean IoU. This suggests that training with a more diverse visual context supports better generalization and improves the model’s ability to distinguish small objects from background interference and speckle noise. A possible explanation is the regularization effect of data diversity: supplementing the training set with more complex or challenging cases forces the model to learn more general, noise-resilient feature representations. As a result, the model avoids overfitting to the details of a single class and becomes more resilient to SAR signal variations and disturbances.

### Impact of SAR acquisition geometry on small vehicle detection

Prior studies show that object recognition performance in SAR imagery is strongly conditioned by geometric factors such as incidence angle and target orientation with respect to the sensor. Pulella et al.^58^ explicitly analyze class performance across incidence/target-orientation distributions and illustrate strong sensitivity to those geometric factors for aircraft. However, for small ground objects (e.g., cars) at our working resolutions it is often impractical to infer front–rear orientation reliably from single-look SAR amplitude: aspect cues are weak, speckle and layover obscure fine structure, and vehicle pose relative to the sensor is ambiguous. Therefore, an explicit heading-angle study for cars would likely be noisy and of limited credibility, whereas our current focus on robustness across clutter/backdrops and acquisition variability is better aligned with operational use.

## Conclusion

This study evaluated the effectiveness of three YOLO architectures – YOLOv7, YOLOv8, and YOLOv12 – for the task of detecting small vehicles in SAR imagery. The experiments incorporated data from diverse sources, including the publicly available SIVED dataset (airborne SAR) and a newly developed satellite SAR dataset based on ICEYE and CAPELLA imagery. Additionally, the impact of speckle noise reduction via image filtering was analyzed.

The results demonstrate that YOLO-based detectors are highly competitive with more complex, rotation-aware detection frameworks. Notably, YOLOv8 achieved the highest detection performance on unfiltered SAR data, showing strong resilience to speckle noise. YOLOv12, although more dependent on preprocessing, outperformed YOLOv7 after applying the Lee filter, confirming the benefits of selective noise reduction for attention-based architectures. These findings underscore the importance of aligning model architecture and filtering strategy with the specific characteristics of the SAR data and the operational use case.

Furthermore, the experiments showed that increasing the diversity of object classes in the training set can enhance detection performance for small objects, likely due to improved generalization and robustness to signal variation and clutter.

The evaluation also highlights that no single metric can fully capture model performance. While metrics like precision and recall are important, detailed analysis of false positives and false negatives is essential to adapt detection systems to real-world applications, where the cost of a missed detection or false alarm may vary significantly.

Future work should explore the integration of orientation-aware mechanisms with lightweight detection architectures, and focus on building high-quality datasets containing small objects in satellite SAR imagery – an area that remains underrepresented despite its growing importance in both civilian and defense applications.

## Data Availability

The datasets generated and analysed during the current study are available in the „VehicleDetection” repository, https://github.com/KK-MUT/VehicleDetection after submission to the corresponding author on reasonable request.
